# Modulation of the Gut–Liver Axis and Nrf2/HO-1-Mediated Antioxidant Defense by *Styela plicata* Extract Against Alcoholic Liver Injury

**DOI:** 10.3390/antiox15040480

**Published:** 2026-04-13

**Authors:** Qiuzhe Li, Yuanyuan Liu, Shuo Shan, Yuxi Wen, Xue Wu, Siquan Xie, Bin Liu, Chao Zhao, Weichao Chen

**Affiliations:** 1 College of Food Science, Fujian Agriculture and Forestry University, No. 15 Shangxiadian Road, Fuzhou 350002, China; 2 College of Marine Sciences, Fujian Agriculture and Forestry University, No. 15 Shangxiadian Road, Fuzhou 350002, China; 3 Nutrition and Bromatology Group, Department of Analytical and Food Chemistry, Faculty of Sciences, Universidade de Vigo, 32004 Ourense, Spain

**Keywords:** *Styela plicata*, alcoholic liver injury, gut–liver axis, antioxidant, Nrf2/HO-1 pathway

## Abstract

*Styela plicata*, an edible ascidian rich in diverse bioactive constituents, represents a promising source of marine natural products for therapeutic discovery. Here, bioactive components from a 95% ethanol extract of *S. plicata* (ESP) were characterized by HPLC-MS/MS, showing that the major constituents were oxygenated small molecules dominated by fatty acyls and carboxylic acid derivatives. In a mouse model of alcohol-induced liver injury, H-ESP treatment (300 mg/kg) significantly reduced serum levels of AST, ALT, and TG (*p* < 0.01), while effectively ameliorating pathological changes in liver tissue, reducing lipid accumulation and inflammatory responses. Transcriptome sequencing (H-ESP vs. model group) identified 1097 differentially expressed genes (172 upregulated and 925 downregulated), and KEGG analysis highlighted significant enrichment of the Toll-like receptor signaling pathway. ESP modulated hepatic metabolite expression, suppressed inflammation via TLR-4/NF-κB pathway inhibition, and boosted antioxidant defenses by activating Nrf2/HO-1 signaling, which was further confirmed by RT-qPCR and immunohistochemistry. ESP increased intestinal SCFAs (acetate, propionate, isobutyrate; *p* < 0.05), improved α-diversity and the *Firmicutes*/*Bacteroidetes* ratio, reversed shifts in *Lactobacillus* and *Bifidobacterium*, and partly restored *Odoribacter*, supporting a gut–liver axis mechanism. Overall, these findings indicate that ESP exerts hepatoprotective effects by modulating the gut–liver axis, and they provide insights for developing natural therapeutics against alcoholic liver disease.

## 1. Introduction

Alcoholic beverages remain widely consumed worldwide, and chronic excessive alcohol intake represents a major global health concern. As the central organ responsible for ethanol metabolism, the liver is particularly vulnerable to alcohol-induced metabolic disruption and cellular injury. Persistent alcohol exposure drives a spectrum of pathological alterations collectively defined as alcoholic liver disease (ALD), ranging from steatosis and alcoholic hepatitis to fibrosis, cirrhosis, and ultimately, hepatocellular carcinoma [1,2,3]. Despite advances in clinical management, effective preventive strategies targeting early pathogenic mechanisms of ALD remain limited.

The pathogenesis of ALD is multifactorial, including hepatotoxicity caused by ethanol and its catabolism, oxidative stress, and the alteration in intestinal microbiota [4,5,6]. Under excessive or long-term alcohol consumption, the levels of ethanol in the body are significantly increased, and alcohol dehydrogenase (ADH), catalase (CAT), and microsomal enzyme system (CYP2E1) are all activated [7,8]. As a metabolite of ethanol, acetaldehyde is further metabolized to acetate to produce nicotinamide adenine dinucleotide (NADH), which leads to mitochondrial oxidation and causes the accumulation of reactive oxygen species (ROS) [9,10,11]. Meanwhile, excessive alcohol consumption disrupts the Nrf2 gene, causing oxidative stress and liver damage [12]. ALD is closely associated with gut microbiota dysbiosis [13]. Ethanol is metabolized in the intestine and produces reactive oxygen species, which triggers an inflammatory response and leads to intestinal barrier dysfunction [14]. Microbial-derived pathogenic components translocate from the intestinal compartment to the hepatic circulation, ultimately inducing hepatocellular injury [15,16]. Consequently, these processes amplify hepatic inflammation and accelerate ALD progression.

In parallel, increasing evidence highlights the microbiota–gut–liver axis as a key regulator of liver disease progression. Alcohol consumption disrupts intestinal barrier integrity and microbial composition, facilitating the translocation of microbial products into portal circulation and promoting hepatic inflammation [17,18]. Experimental studies further demonstrate that gut microbiota dysbiosis contributes to oxidative stress-mediated liver injury, whereas microbiota-derived metabolites participate in host redox regulation and immune signaling [19,20]. In particular, short-chain fatty acids (SCFAs) produced by gut microbes have been shown to modulate hepatic inflammation, oxidative stress, and metabolic homeostasis in liver disease models, supporting antioxidant modulation of the gut–liver axis as a promising therapeutic strategy [21,22,23].

At present, bifendate remains a first-line therapeutic option for ALD. However, this kind of hepatoprotective drug has side effects such as drug withdrawal rebound. Therefore, intervention with natural active compounds (prebiotics, polyphenols, flavonoids, alkaloids, and terpenoids) is a potential way to effectively prevent alcoholic liver injury [15,24,25,26]. Recent studies highlight the therapeutic potential of medicinal plants and their bioactive constituents in oxidative stress-related disorders. Plant-derived phenolics and flavonoids modulate redox and inflammatory signaling pathways; for example, ethyl gallate and quercetin have exhibited antioxidant and organ-protective effects in experimental models [27,28,29], and systematic analyses further support the broad pharmacological relevance of ethnomedicinal plants. Beyond terrestrial plants, marine organisms provide structurally diverse bioactive compounds. In this context, *Styela plicata*, an edible ascidian widely distributed in temperate coastal waters, represents a promising marine candidate for investigating antioxidant and hepatoprotective mechanisms [30]. Beyond its ecological role, *S. plicata* is increasingly recognized as a “marine functional food” due to its exceptional nutritional profile, being rich in essential amino acids, high-quality polyunsaturated fatty acid, and minerals. It has the effects of immune regulation, anti-tumor, antioxidant, anti-inflammatory and liver protection [31,32]. However, whether *S. plicata* exerts protective effects through antioxidant signaling mechanisms linked to gut–liver interactions during alcohol-induced liver injury remains largely unexplored. Based on these considerations, we hypothesized that ethanol extract from ESP alleviates alcohol-induced liver injury primarily through modulation of antioxidant signaling and restoration of gut–liver axis homeostasis. Specifically, we propose that ESP activates the Nrf2/HO-1 antioxidant pathway while reshaping gut microbiota composition and associated metabolic outputs, thereby reducing inflammatory signaling and oxidative damage. To test this hypothesis, we integrated biochemical assessment, transcriptomics, metabolomics, microbiome profiling, and short-chain fatty acid analysis to construct a multi-level mechanistic framework linking antioxidant regulation with microbiota-mediated hepatoprotection.

## 2. Materials and Methods

### 2.1. Preparation and Component Analysis of Bioactive Components of S. plicata (ESP)

Fresh *S. plicata* were collected from the coastal waters of Zhangzhou, Fujian Province, China. After washing with water to remove surface and symbiotics, the samples were dehydrated, dried, and crushed for further use. *S. plicata* dry powder was added to 95% ethanol at a ratio of 20:1 (V/W) and extracted under ultrasonic (45 Hz, 350 W) conditions for 40 min. After filtration with four layers of gauze, the mixture was centrifuged (4000 rpm, 10 min, 25 °C). The supernatant was then collected and filtered to obtain the final filtrate. The filtrate was steamed at 45–60 °C under reduced pressure until it had no ethanol flavor. Finally, ESP was obtained by vacuum freeze-drying [33].

### 2.2. Animal Experiments

Forty-eight specific pathogen-free (SPF) male ICR mice (6 weeks old, weighing 20–25 g) were obtained from the Animal Experiment Center of Fuzhou General Hospital. They were kept at a constant temperature of 22–26 °C, and a light/dark cycle was maintained at 12 h intervals under controlled environmental conditions. Eight mice were initially allocated to each group, and the number of animals included in specific analyses is indicated in the corresponding figure legends. After acclimatizing for two weeks, the mice were randomly divided into NC, MC, PC (Bifendate, 150 mg/kg), L-ESP (ESP 75 mg/kg), M-ESP (ESP 150 mg/kg), and H-ESP (ESP 300 mg/kg) groups, with eight mice in each group. All experimental groups received alcohol gavage 4 h post-administration of bifendate and ESP, with the NC group serving as the untreated control. The mice were given 35% alcohol for the first two weeks, and then the alcohol concentration was increased by 5% every week until it reached 50%. The dose of alcohol by gavage was 12 mL/kg for seven weeks [34]. Body weight and general health status were monitored throughout the experimental period. At the end of treatment, mice were anesthetized prior to blood collection and tissue harvesting. Liver tissues were rapidly excised, weighed, and processed for biochemical, histological, and molecular analyses.

All animal experiments were conducted in accordance with the Guiding Principles for Research Involving Animals and Human Beings and complied with the welfare requirements of China’s national standard GB/T 42011-2022 [35] (General Rules for Laboratory Animal Welfare). The study protocol was reviewed and approved by the Ethics Committee of Fujian Agriculture and Forestry University (approval number: PZCASFAFU21026).

### 2.3. Analysis of Biochemical Index and Histopathological Analysis

At the end of the experiment, the mice were fasted overnight, anesthetized with isoflurane, and euthanized by cervical dislocation. Blood and tissues were collected immediately. Under anesthesia, mouse blood samples were collected via vacuum aspiration and subsequently centrifuged (3000 rpm, 15 min, 4 °C) to isolate the serum. The contents of AST, ALT, and TG in the serum were quantified according to the operation methods of the kits (Nanjing Jiancheng Bioengineering Institute, Nanjing, China). Additionally, liver tissues designated for molecular/omics analyses were snap-frozen in liquid nitrogen and stored at −80 °C.

Histopathological analysis (H&E): After euthanasia, livers were excised and gently rinsed with sterile normal saline, then fixed in 10% neutral-buffered formalin. Tissues were dehydrated, cleared, paraffin-embedded, and sectioned using a rotary microtome. Sections were floated on a 40 °C water bath and dried at 60 °C for 3 h. After deparaffinization in xylene and rehydration through a graded ethanol series (100%, 95%, 80%, 70%) to distilled water, sections were stained with hematoxylin (5–10 min), differentiated in 0.1% acid alcohol (10–30 s), blued in PBST (30–60 s), counterstained with eosin (30–120 s), dehydrated and cleared, and mounted with neutral resin. The slides were examined and imaged under a light microscope.

Immunohistochemistry (IHC): Paraffin sections were deparaffinized and rehydrated as described above. After antigen retrieval, sections were washed with immunostaining buffer, and endogenous peroxidase activity was quenched using 3% H_2_O_2_ in methanol for 15 min. Sections were then blocked for 45 min and incubated with primary antibodies at 4 °C overnight. The next day, sections were equilibrated to room temperature for 1 h, washed, and incubated with an HRP-conjugated secondary antibody for 45 min at room temperature. Color development was performed using a chromogenic substrate, followed by hematoxylin counterstaining (5–10 min). Sections were differentiated briefly in acid alcohol, blued in PBST, dehydrated through graded ethanol, cleared in xylene, and mounted with neutral resin. Images were acquired under a light microscope, and staining intensity was quantified using Image-Pro Plus 6.0.

Additionally, liver tissues designated for molecular/omics analyses were snap-frozen in liquid nitrogen and stored at −80 °C.

### 2.4. RNA-Seq Analysis

The H-ESP and MC groups were selected for transcriptome sequencing. The specific operation process was as follows: Total RNA was extracted, and polyadenylated transcripts were enriched. These transcripts were converted to double-stranded cDNA and prepared for sequencing through end repair, A-tailing, and adapter ligation. Size-selected fragments were PCR-amplified, with final library validation preceding Illumina sequencing.

### 2.5. RT-qPCR and Immunohistochemistry

Liver RNA was reverse-transcribed into cDNA using the PrimeScript™ RT kit (Thermo Fisher Scientific, Waltham, MA, USA). The primers of TLR-4/Nrf2/HO-1/NF-α/NF-κB in mice were designed according to the NCBI primer design principles, following the instructions of the NovoStart SYBR qPCR super kit (Suzhou, China). Amplification was performed using the ABI 7300 PCR system (Applied Biosystems, Foster City, CA, USA). Target gene expression levels were subsequently quantified. Fresh mouse livers were washed and treated with paraffin embedding. After slicing, spreading, baking, dewaxing and hydration, the wax blocks were washed with immunostaining solution. The tissue samples were outlined with an immunohistochemical oil pen, and then subjected to enzyme elimination, washing, and sealing. This was followed by overnight incubation with primary antibodies. After thorough washing with TBST, the samples were incubated with secondary antibodies, washed, and subjected to color development, restaining, decolorization, and termination. After anti-bluing, differentiation, rinsing and soaking, the wax blocks were finally sealed with neutral gum. They were examined and filmed under a light microscope. The fluorescence intensity of immunohistochemical staining was calculated by Image-Pro plus 6.0 analysis.

### 2.6. LC-MS Metabolomics of Liver

Lyophilized liver samples from the NC, MC and H-ESP groups were processed, and 80 μL of supernatant was collected after preparation. Aliquots from all prepared samples were pooled in equal volumes to create a composite QC sample, which was then subdivided into six identical portions. These QC replicates were analyzed intermittently throughout the sequencing run to monitor instrument precision and stability. Before sampling, 3 QC samples were used to monitor the precision of the instrument. An additional QC sample was processed after every six experimental samples to evaluate analytical stability. Using liquid chromatogram–mass spectrometry, AnalysisBase File Converter converted the original data format. After pre-processing on MSDIAL software (v.4.9) platform, the sample features were further screened, and a visualization matrix was obtained.

### 2.7. 16S rRNA Gene Sequencing and Determination of SCFAs

Genomic DNA was isolated from colonic microbiota using the OMEGA-Soil DNA Kit (Omega Bio-Tek, Inc., Norcross, GA, USA) following the manufacturer’s protocol. The DNA was then subjected to paired-end sequencing using the Illumina Miseq PE300 (San Diego, CA, USA) high-throughput sequencing platform. SCFAs were analyzed qualitatively and quantitatively by gas chromatography–mass spectrometry (GC-MS).

### 2.8. Statistical Analysis

For the animal experiments, each group contained 8 mice (n = 8). Data are presented as the mean ± standard deviation (Mean ± SD). GraphPad Prism 8 and SPSS software (v.31.0) were used for plotting and statistical analyses. Differences among multiple groups were assessed by one-way analysis of variance (one-way ANOVA), followed by Tukey’s post hoc test. A value of *p* < 0.05 was considered statistically significant.

## 3. Results and Discussion

### 3.1. Analysis of ESP

The active components of *S. plicata* were extracted with 95% ethanol solution (V/W), with subsequent phytochemical characterization performed via high-performance liquid chromatography–tandem mass spectrometry (HPLC-MS/MS). The main compounds in ESP are oxygenated small molecules, including organooxygen compounds and their subclasses, such as carboxylic acids and derivatives and fatty acyls, together with benzene/substituted benzene derivatives. Several metabolites, including dihydrocoumarin, lipoxin A4, and puerarin, were putatively annotated based on MS/MS spectral similarity (Table 1). Database matching also suggested features structurally related to compounds, such as irbesartan and perindopril; however, these annotations were not confirmed using authentic reference standards and should therefore be considered tentative.

Previous studies have reported antioxidant or hepatoprotective activities for compounds belonging to similar chemical classes, suggesting that the biological effects of ESP may be associated with the combined action of multiple bioactive constituents rather than individual molecules. To better interpret the relationship between the putatively annotated metabolites in ESP and the observed bioactivities, we focused on several representative candidates with prior experimental evidence for hepatoprotection or antioxidant modulation. Puerarin and related phytochemicals have been reported to exert protective effects against alcoholic liver injury [16,36,37]. Lipoxin A4, a pro-resolving lipid mediator, has also shown protective effects in murine alcoholic hepatitis by limiting hepatic inflammation [38]. Taken together, these studies support a biologically plausible link between the chemical classes detected in ESP and the antioxidant/hepatoprotective phenotypes.

### 3.2. Protective Effect of ESP on Alcoholic Liver Injury in Mice

**Figure 1:**
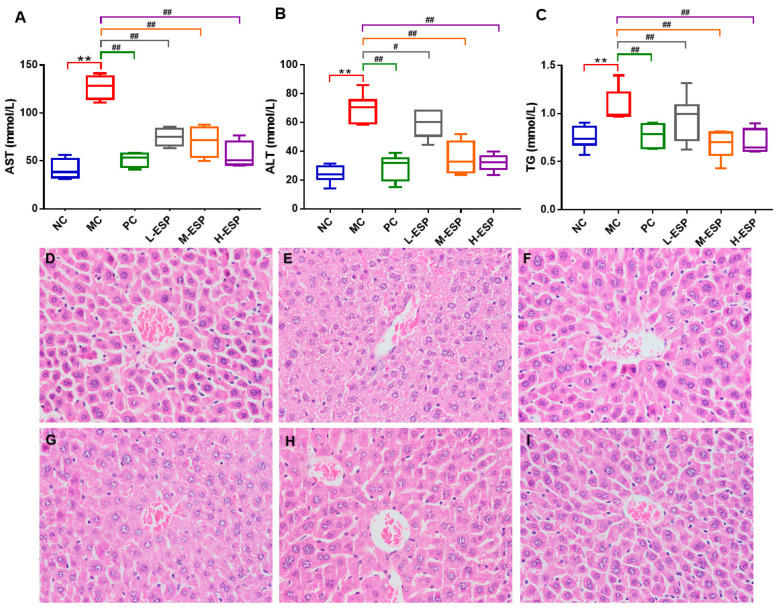
Changes in mice from different groups (n = 6 per group for statistical analysis) and histopathological analysis of liver (400× magnification). (**A**) AST; (**B**) ALT; (**C**) TG; (**D**) NC; (**E**) MC; (**F**) PC; (**G**) L-ESP; (**H**) M-ESP; (**I**) H-ESP. Compared with NC group, ** *p* < 0.01; compared with MC group, ## *p* < 0.01, # *p* < 0.05.

To determine whether ESP exerts protective effects against alcohol-induced liver injury, we first assessed serum biochemical markers and hepatic histopathology. SPF male ICR mice (6 weeks old; n = 8/group) were subjected to a 7-week alcohol gavage protocol (12 mL/kg; 35% ethanol for 2 weeks, then increased by 5% weekly to 50%). ESP (75/150/300 mg/kg) or bifendate (150 mg/kg) was administered once daily, and alcohol was gavaged 4 h later. The model control (MC) group exhibited markedly elevated serum concentrations of TG (*p* < 0.01), ALT (*p* < 0.01), and AST (*p* < 0.01) compared to the normal control (NC) group, indicating successful induction of hepatic metabolic dysfunction and confirming that the model was established successfully. ESP treatment dose-dependently reduced these biochemical indicators, with the 300 mg/kg group (H-ESP) showing the most pronounced improvement, approaching levels observed in the NC group. Consequently, this concentration was used for subsequent experiments (Figure 1A–C). Importantly, no mortality or signs of overt toxicity were observed in the ESP-treated mice throughout the study period. Body weight remained stable across groups, and no gross pathological abnormalities were detected upon necropsy, indicating that ESP was well tolerated under the present experimental conditions. Comparative analysis revealed comparable hepatoprotective efficacy between ESP and the positive control bifendate (PC), with no statistically significant differential therapeutic outcomes observed. Elevated ALT, AST, and TG levels are key indicators of liver health, and lowering them is widely considered crucial for preventing and managing alcohol-related liver damage [31,32,36]. Histopathological study of the liver can directly identify lesions on the liver tissue, which is another important index for alcoholic liver injury [31,32]. Liver specimens were processed for histological evaluation through hematoxylin–eosin (H&E) staining, enabling morphological assessment of hepatic architecture and pathological alterations (Figure 1D–I). In the NC group, the liver tissue of mice exhibited intact nuclei, with hepatic cords arranged in a uniform radial pattern, and no signs of pathological alterations (Figure 1D). Hepatocytes showed significant pathological changes in the MC group, manifested as incomplete cell structure, cytoplasmic swelling, abnormal cell nucleus morphology, the disappearance of radial hepatic cord morphology, disorderly arrangement, and intracellular lipid droplet accumulation (Figure 1E). After treatment with bifendatatum, the mice liver tissue had a relatively complete cell structure, no abnormal nucleus morphology, normal hepatic cord distribution, and the overall morphology was relatively close to that of the NC group. Simultaneously, the ESP-treated group displayed well-organized hepatic cords, normal cellular morphology, and minimal lipid droplet accumulation (Figure 1G–I), with the H-ESP group showing the most obvious effects. The above results indicate that ESP reduced the contents of ALT, AST, and TG, and treatment significantly attenuated alcohol-induced hepatic histopathological alterations in mice, demonstrating its protective effect against alcoholic liver injury.

### 3.3. Identification of ESP-Regulated Genes and Pathways by Transcriptome Sequencing

**Figure 2:**
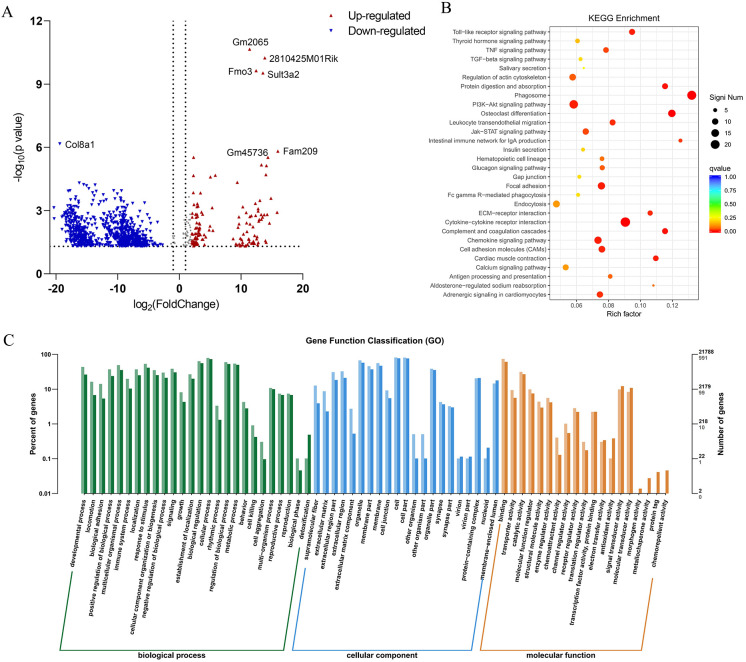
Differentially expressed genes (DEGs) in liver. (**A**) Volcano map of DEGs between H-ESP/MC groups; (**B**) KEGG enrichment of DEGs in H-ESP/MC groups; (**C**) GO terms of DEGs in H-ESP/MC groups (The dark-colored bars represent all genes, while the light-colored bars represent differentially expressed genes).

To further explore the molecular mechanisms underlying the hepatoprotective effects of ESP, transcriptomic sequencing was performed to identify differentially expressed genes between the H-ESP and model groups. RNA-seq analysis of hepatic tissue revealed 1097 differentially expressed genes (DEGs) between the H-ESP-treated and MC mice, comprising 172 upregulated and 925 downregulated transcripts (Figure 2A). According to the GO terms, in biological processes, the predominant enrichments were observed for cellular processes, biological regulation, and metabolic processes. Notably, in the CC category, a significant proportion of gene products were located in the “membrane” and “extracellular region,” implying that ESP may affect membrane receptors and cell–matrix interactions (Figure 2C). KEEG pathway analysis demonstrated significant enrichment of differentially expressed genes, most notably the Toll-like receptor pathway. The PI3K-Akt, Chemokine, and TNF signaling pathways also exhibited substantial gene enrichment patterns (Figure 2B). A total of 11 Toll-like receptors have been identified in humans, among which TLR-4 mainly mediates LPS signaling. TLR-4 expression levels have been reported to be significantly elevated in ALD patients and higher in AH patients [31,32,36]. Hence, ESP’s therapeutic mechanism likely involves suppression of TLR-4 and TLR-2 gene expression, thereby modulating both the Toll-like receptor signaling cascade and its downstream NF-κB pathway, ultimately inhibiting the inflammatory response induced by alcohol intake.

### 3.4. Effects of ESP on Related Genes and Immunohistochemistry of Liver

**Figure 3:**
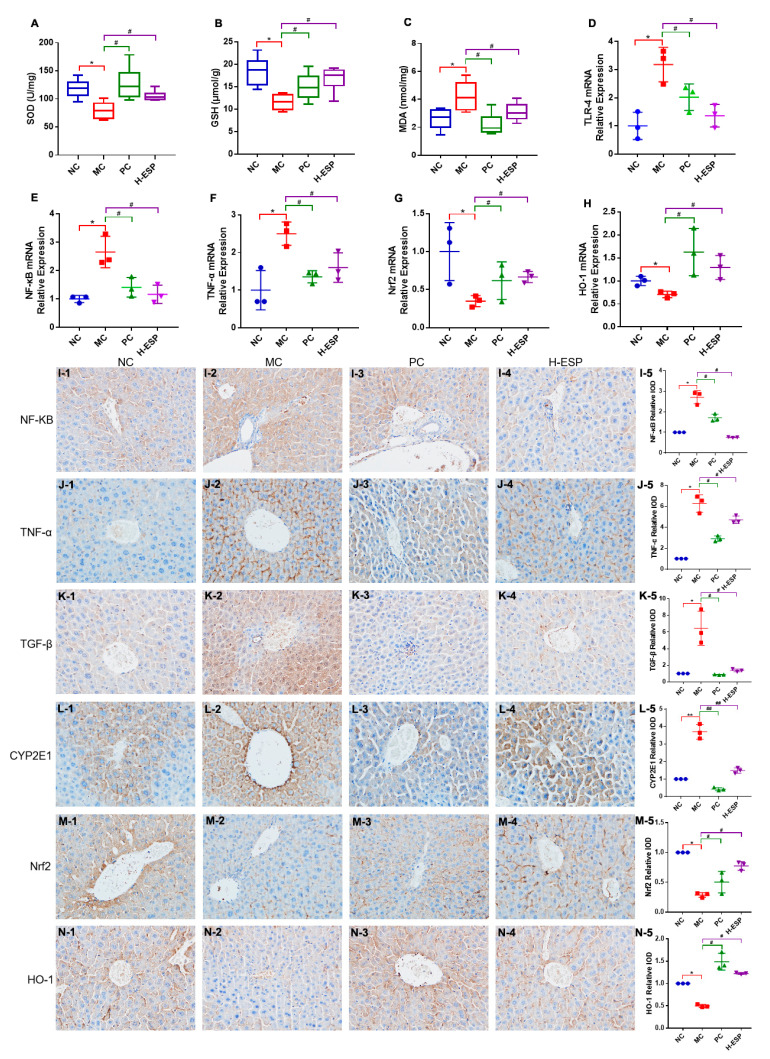
The contents of SOD (**A**), GSH (**B**), and MDA (**C**) in liver homogenate (n = 6). Related mRNA (**D**–**H**) (n = 3) and protein expressions (**I**–**N**) in liver. Compared with NC group, ** *p* < 0.01, * *p* < 0.05; compared with MC group, ## *p* < 0.01, # *p* < 0.05.

Given that KEGG enrichment highlighted inflammation- and oxidative stress-related pathways, we next evaluated key antioxidant markers and conducted immunohistochemical analysis to verify the molecular effects of ESP on liver tissues. The PC and H-ESP treatments restored antioxidant defenses, with superoxide dismutase (SOD) and glutathione (GSH) concentrations approaching normal physiological levels. These values were significantly elevated relative to the MC group (*p* < 0.05) (Figure 3). The malondialdehyde (MDA) content in the H-ESP group was significantly reduced by 25% compared with the MC group (*p* < 0.05) and the NC group (ns) (Figure 3A–C). A study reported that alcohol intake inhibits antioxidant activity and causes lipid peroxidation in the liver, which leads to oxidative stress [31]. In this work, ESP had a significant regulatory effect on SOD, GSH and MDA contents in the liver, which suggests that the antioxidant capacity of the liver was significantly restored.

RT-qPCR and immunohistochemistry were used to examine key molecular pathways. The H-ESP treatment group showed marked transcriptional changes in hepatic tissue compared to the model controls, with significantly reduced expression of pro-inflammatory mediators (TLR-4, NF-κB, TNF-α) and elevated levels of antioxidant genes (Nrf2, HO-1) (*p* < 0.05) (Figure 3D–H). Immunohistochemistry further confirmed that the protein expression patterns of NF-κB, TNF-α, Nrf2, and HO-1 were consistent with the RT–qPCR results. In addition, while TGF-β and CYP2E1 expression levels were elevated in the MC group’s liver tissue, H-ESP treatment markedly reduced these levels to near-normal values comparable to the NC group (Figure 3I–N).

The inflammatory mechanism underlying ALD likely involves TLR-4 and TGF-β suppression coupled with NF-κB activation, leading to upregulated TNF-α expression and subsequent systemic inflammation [39,40]. The high expression of CYP2E1 was correlated with oxidative stress in the alcohol-induced liver injury [11]. Perennial drinking can disrupt the antioxidant-related gene Nrf2, further inhibiting the expression of antioxidant oxidase HO-1, and resulting in elevated oxidative stress levels and liver damage [41]. The experimental data suggest that ESP effectively inhibited the upregulation of TLR-4 and TGF-β triggered by excessive LPS and alcohol consumption, thereby suppressing NF-κB activation and reducing TNF-α levels. This mechanism contributed to the mitigation of inflammatory reactions and prevention of ECM overproduction, ultimately protecting hepatic tissue. Furthermore, ESP enhanced the body’s antioxidant defense system by modulating CYP2E1 expression and stimulating the Nrf2/HO-1 signaling pathway, which further supported its hepatoprotective function.

### 3.5. ESP Improved the Metabolism of the Liver

**Figure 4:**
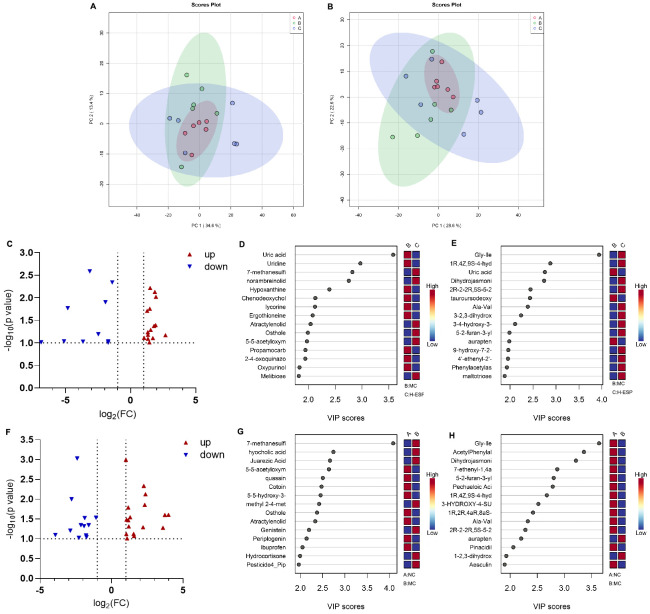
The metabolism of the liver in alcohol-induced liver injury mice. Principle component analysis score plot of liver metabolites in positive mode (**A**) and negative mode (**B**). (**C**–**E**) Comparison of liver metabolites between the MC and H-ESP groups. (**F**–**H**) Differential liver metabolites in the NC and MC groups.

To further investigate alterations in hepatic metabolism, liver metabolites were profiled using LC-MS–based metabolomics. According to principal component analysis, there was obvious clustering of the NC, MC and H-ESP groups in both the positive and negative modes. A discernible separation trend was observed, with partial overlap among the clusters, indicating group-associated differences in hepatic metabolite profiles (Figure 4A,B). Comparing between the MC and H-ESP groups identified 25 differential metabolites (Figure 4C–E), including uric acid, aurapten, Gly-Ile, and lycorine. Comparing between the MC and NC groups identified 55 different metabolites (Figure 4F–H), including juarezic acid, hyocholic acid, L-alpha-phosphatidylethanolamine, aurapten, dihydrojasmonic acid, Gly-Ile, aesculin, pechueloic acid, schisandrin A, and quassin. In conclusion, the liver metabolites of mice with alcoholic liver injury were changed with ESP treatment, among which the differential metabolites screened can be used as candidate biomarkers for treating or evaluating the development of ALD progression.

### 3.6. ESP Modulates the Abundance and Diversity of Gut Microbiota

**Figure 5:**
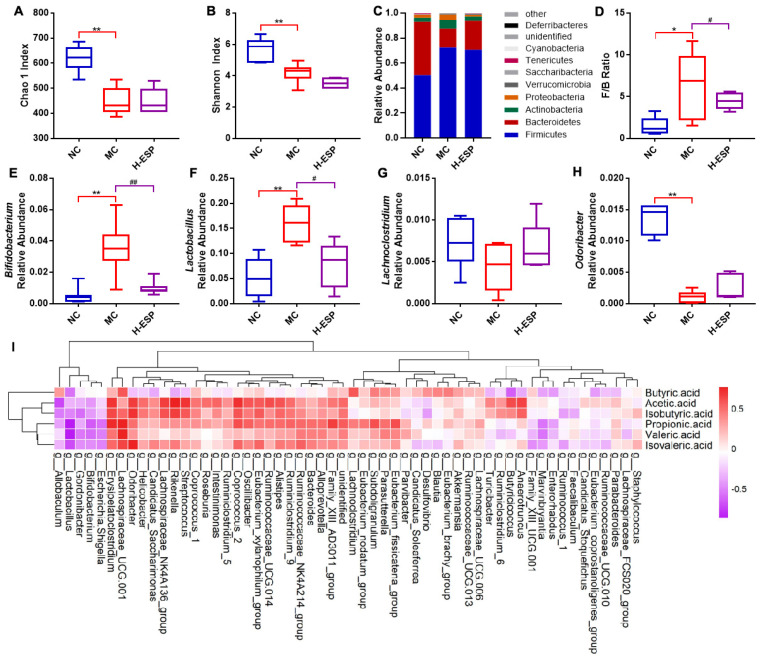
Changes in the gut microbiota profile of mice colon. (**A**,**B**) Chao 1 and Shannon diversity indices. (**C**) Phylum-level distribution of intestinal microbial communities. (**D**) The proportion of Firmicutes relative to Bacteroidetes. (**E**–**H**) Four highly abundant bacterial genera identified at the genus level. (**I**) Statistical analysis of Spearman’s correlations between the genera of colon microbiota and SCFAs. Compared to the NC group, ** *p* < 0.01, * *p* < 0.05; compared to the MC group, ## *p* < 0.01, # *p* < 0.05.

Considering the known contribution of the gut–liver axis to alcoholic liver injury, we subsequently analyzed gut microbiota composition to determine whether ESP modulates intestinal microbial structure. The Chao species richness index (Figure 5A) and Shannon diversity index (Figure 5B) showed that H-ESP augmented the richness and biodiversity of the colonic microbiota in mice with alcoholic liver injury. Mouse colon bacteria were dominated by *Firmicutes, Bacteroidota, Actinobacteriota* and *Proteobacteria*. Among these, *Firmicutes* and *Bacteroidota* were the dominant phyla in mouse colon, comprising over 85% of the entire gut microbiome (Figure 5C). In the NC and H-ESP groups, the F/B ratio was substantially reduced relative to that in the MC group. (*p* < 0.05). It was reported that the intestinal health of mice was negatively correlated with the F/B ratio [42]. At the genus level, H-ESP notably attenuated the prevalence of *Bifidobacterium* and *Lactobacillus* and enhanced the abundance of *Lachnoclostridium* and *Odoribacter* (Figure 5D–H). *Bifidobacterium* are generally considered as probiotics. However, it was shown that the high prevalence of Bifidobacterium in ASD patients was related to constipation, which is also a common symptoms in ALD patients [43]. The low abundance of *Lachnoclostridium* and *Odoribacter splanchnicus* is typically associated with the onset of intestinal inflammation, a condition linked to butyric acid deficiency [44].

GC-MS was employed to quantify the level of SCFAs within the intestinal contents. Compared with the MC group, acetic acid, propionic acid, isobutyric acid, isovaleric acid, valeric acid in the H-ESP group were significantly increased (*p* < 0.05), indicating that H-ESP could facilitate the enrichment of SCFAs within the murine intestine (Figure S1). *Faecalibaculum* populations demonstrated decreased abundance with higher isobutyric and acetic acid concentrations, while showing increased abundance alongside elevated valeric acid. While the relative abundance of *Enterorhabdus* showed an inverse relationship with butyric acid and propionic acid, *Lachnoclostridium* demonstrated a direct positive correlation with these compounds. *Alistipes* abundance showed a significant positive association with acetic acid, propionate, isobutyrate, valeric acid, and isovaleric acid levels (Figure 5I). Propionic acid was reported to markedly attenuate cholesterol levels and suppress the elevation of inflammatory cytokines during the progression of alcoholic liver injury [45]. In addition, butyric acid plays a crucial role in modulating mucin and tight junction protein expression, thereby preventing intestinal barrier dysfunction induced by excessive alcohol consumption [46]. Notably, despite the low content of n-valerate, there is evidence that valerate may be more efficacious in provoking an anti-inflammatory response because it is less toxic than butyrate and more effective than acetic acid [47]. Studies have revealed that *Alistipes* may regulate the intestinal pH and immune microenvironment by generating SCFAs, including acetic acid [48,49,50]. Taken together, these findings indicate that ESP-induced microbiota remodeling is accompanied by enhanced SCFA production, which may contribute to attenuation of hepatic inflammation and oxidative stress within the gut–liver axis framework.

### 3.7. Gut–Liver Axis Underlying the Protective Effects of ESP

**Figure 6:**
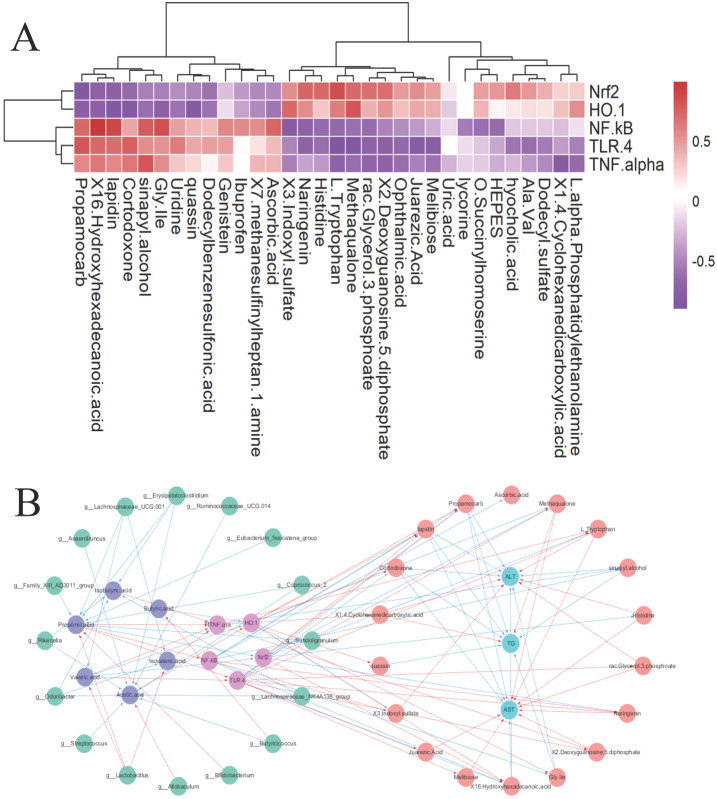
(**A**) Hierarchical cluster analysis using Spearman’s rank correlation coefficients was conducted to examine relationships between glucose-regulating parameters and cecal microbial genera, where color gradients represent correlation strength. (**B**) A correlation network was constructed to visualize partial correlation relationships among gut microbial taxa, genes, differential metabolites and SCFAs (the red line indicates negative correlations; the blue line indicates positive correlations).

Based on the enrichment results highlighting inflammatory signaling, we next examined key proteins involved in the TLR4–NF-κB and Nrf2/HO-1 pathways to validate the transcriptomic findings at the molecular level. The genes Nrf2 and HO-1 were positively correlated with Naringenin, which has been demonstrated to exert hepatoprotective effects in animal models of acute liver injury [51]. 3-Indoxylsulfate, 2′-Deoxyguanosine 5′-diphosphate, L-tryptophan, and methaqualone were identified. Among them, 3-Indoxylsulfate can activate the aryl hydrocarbon receptor, thereby improving alcoholic liver injury and inhibiting mold growth. These metabolites were negatively correlated with 16-Hydroxyhexadecanoic acid, Propamocarb, Cortodoxone, Gly Ile, Lapidion, and sinaphyl alcohol [52]. However, the gene NF-κB, TLR-4, and TNF-α had opposite correlations with above liver metabolites. Correlation analysis was carried out between mouse liver genes and differential metabolites (Figure 6A). The visual association diagram shows the associations between gut microbiota, SCFAs, liver genes, metabolites, and biochemical parameters (Figure 6B). TG and ALT exhibited strong positive correlations with Propamocarb, Cortodoxone, Lapidin, sinapyl alcohol, and Gly-Ile, and a strong negative correlation with 1,4-cyclohexanedicarboxylic acid. AST shows a strong positive correlation with 16-hydroxyhexadecanoic acid but strong negative correlations with histidine and L-tryptophan, naringenin, 2′-deoxyguanosine 5′-diphosphate, juarezic acid, melibiose, and methaqualone. It has been reported that histidine can improve liver oxidative and inflammatory damage induced by alcohol due to its high antioxidant activity [53]. These results reveal coordinated associations among gut microbiota, SCFAs, hepatic gene expression, and metabolic alterations, suggesting potential interactions within the gut–liver axis.

### 3.8. Integrated Analysis of the Gut–Liver Axis Mechanism

**Figure 7:**
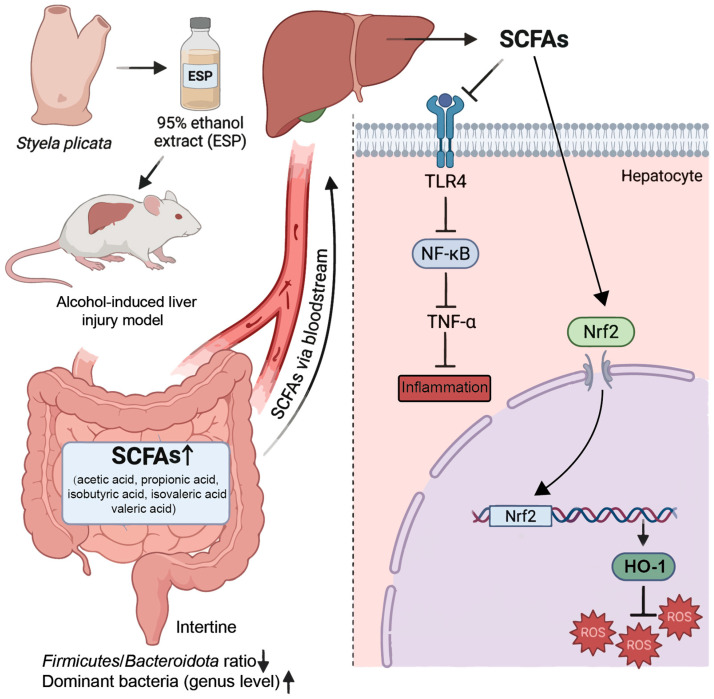
Proposed mechanism of ESP-mediated hepatoprotection via the gut–liver axis.

To integrate the microbiome, transcriptomic, and molecular findings, a conceptual mechanistic model of the gut–liver axis was constructed (Figure 7). This model links gut microbial remodeling with intracellular antioxidant and inflammatory signaling in hepatocytes, providing a systems-level interpretation of the hepatoprotective effects of ESP.

Chronic alcohol exposure resulted in elevated serum AST, ALT, and TG levels, accompanied by hepatic inflammation and lipid droplet accumulation, which are characteristic pathological features of early alcoholic liver injury. ESP treatment significantly attenuated these pathological alterations, suggesting a protective effect against alcohol-induced hepatic damage. At the intestinal level, ESP improved microbial α-diversity and partially restored the *Firmicutes*/*Bacteroidetes* ratio. Notably, beneficial genera, including *Lactobacillus* and *Bifidobacterium*, were increased, while *Odoribacter* showed partial recovery. Alcohol-induced gut microbiota dysbiosis is recognized as a key driver of liver injury through disruption of intestinal barrier integrity and activation of inflammatory signaling along the gut–liver axis [54,55]. The observed microbial remodeling therefore suggests that ESP may mitigate alcohol-associated liver injury partly through modulation of intestinal microbial ecology. These microbial changes were accompanied by significant increases in SCFAs, including acetate, propionate, and isobutyrate. SCFAs are major microbiota-derived metabolites that regulate host metabolic and immune homeostasis and play an important role in gut–liver metabolic communication [56]. The elevation of SCFAs observed in the ESP-treated group therefore supports enhanced microbiota-derived metabolic signaling.

Consistent with the transcriptomic enrichment of the Toll-like receptor pathway, ESP inhibited hepatic TLR4–NF-κB signaling, resulting in decreased NF-κB activation and reduced inflammatory responses. Activation of the TLR4-mediated inflammatory cascade is widely recognized as a central mechanism linking gut-derived endotoxin exposure to hepatic inflammation in alcohol-related liver injury [57]. In parallel, ESP promoted the release of Nrf2 from cytoplasmic inhibition and facilitated its nuclear translocation, leading to increased expression of the downstream antioxidant enzyme HO-1. The Nrf2–HO-1 signaling pathway constitutes a major cellular defense system against oxidative stress and plays a critical role in protecting hepatocytes from ROS-induced damage [58]. Activation of this pathway in the present study was associated with reduced ROS levels and decreased oxidative stress.

Collectively, these results support a coordinated gut–liver axis mechanism in which ESP-induced microbiota remodeling and SCFA production contribute to suppression of hepatic inflammatory signaling and activation of antioxidant defenses. Although the present findings establish an integrated framework linking microbial modulation to intracellular redox regulation, further studies are required to identify the specific bioactive constituents responsible for these effects and to evaluate their translational potential in human alcoholic liver disease. Given that gut dysbiosis and oxidative stress are recognized drivers of early alcohol-associated liver injury, modulation of microbiota–redox interactions by marine-derived bioactive extracts, such as ESP, may represent a promising strategy for early intervention or prevention. As an edible marine organism, Styela plicata may therefore have potential value as a functional food ingredient or nutraceutical candidate, pending further safety evaluation and clinical validation.

## 4. Conclusions

This study confirms that the active components from *S. plicata* not only improve liver function parameters and histopathological features but also regulate hepatic homeostasis by attenuating the TLR-4/NF-κB inflammatory pathway and promoting the activation of the Nrf2/HO-1 antioxidant defense system. Remarkably, ESP’s modulation of gut microbiota composition and associated metabolic products underscores the fundamental importance of the gut–liver axis in the etiology of alcohol-induced liver injury. This dual regulatory mechanism transcends the single-target limitations of conventional hepatoprotective drugs, offering novel perspectives for developing integrated therapeutic strategies rooted in natural products.

## Figures and Tables

**Table 1:** The main compounds in ESP.

Name	Formula	Molecular Weights	PPM	Classification
3,4-Dihydro-2H-1-benzopyran-2-one	C_9_H_8_O_2_	148.05	3.96	3,4-Dihydrocoumarins
Erythritol	C_4_H_10_O_4_	122.05	20.05	Xenobiotics
Lipoxin A4	C_20_H_32_O_5_	352.22	11.72	Fatty acyls
Puerarin	C_21_H_20_O_9_	416.11	4.81	Lipid
6-Shogaol	C_17_H_24_O_3_	276.17	0.57	Benzene and substituted derivatives
Formononetin	C_16_H_12_O_4_	268.07	20.25	Isoflavonoids
Irbesartan	C_25_H_28_N_6_O	428.23	14.55	Benzene and substituted derivatives
Lovastatin	C_24_H_36_O_5_	404.25	15.7	Lactones
Perindopril	C_19_H_32_N_2_O_5_	368.23	17.12	Carboxylic acids and derivatives
(S)-Abscisic acid	C_15_H_20_O_4_	264.13	1.52	Prenol lipids
4-Methylumbelliferone	C_10_H_8_O_3_	176.04	0.13	Coumarins and derivatives
9-cis-Retinoic acid	C_20_H_28_O_2_	300.2	1.71	Prenol lipids
Azelaic acid	C_5_H_9_NO_3_S	188.1	10.57	Fatty acyls
Adipic acid	C_6_H_10_O_4_	146.05	9.93	Fatty acyls
Baicalein	C_15_H_10_O_5_	270.05	26.59	Flavonoids
D-Ribose	C_5_H_10_O_5_	150.05	2.24	Organooxygen compounds
Deoxycholic acid	C_24_H_40_O_4_	392.29	3.06	Steroids and steroid derivatives
Galactitol	C_6_H_14_O_6_	182.07	5.1	Organooxygen compounds
Glycerophosphocholine	C_8_H_21_NO_6_P	258.11	0.95	Glycerophospholipids
Isophorone	C_9_H_14_O	138.1	3.43	Carbonyl compounds
Isoquinoline	C_9_H_7_N	129.05	3.78	Isoquinolines and derivatives
Lipoxin B4	C_20_H_32_O_5_	352.22	14.13	Fatty acyls
Methyl jasmonate	C_13_H_20_O_3_	224.14	3.31	Fatty acyls
Mupirocin	C_26_H_44_O_9_	500.29	29.42	Fatty acyls
N-Acetyl-D-glucosamine	C_8_H_15_NO_6_	221.08	25.13	Organooxygen compounds
Oleic acid	C_18_H_34_O_2_	282.25	2.6	Fatty acyls
Palmitoleic acid	C_16_H_30_O_2_	254.22	6.11	Fatty acyls

## Data Availability

Data are available on request. The RNA-seq data presented in the study are openly available in the FigShare repository at the following DOI: https://doi.org/10.6084/m9.figshare.31931727 (accessed on 8 March 2026).

## Supplementary Materials

The following supporting information can be downloaded at: https://www.mdpi.com/article/10.3390/antiox15040480/s1.
