# Supplementary material for: Modulation of the Gut–Liver Axis and Nrf2/HO-1-Mediated Antioxidant Defense by Styela plicata Extract Against Alcoholic Liver Injury
Source: Antioxidants (Basel). 2026 Apr 13;15(4):480. doi: 10.3390/antiox15040480 (PMC13114156; doi:10.3390/antiox15040480)
Supplement: Supplementary file 1 [file antioxidants-15-00480-s001.zip › antioxidants-4140231-supplementary.pdf]

Supplementary Materials:

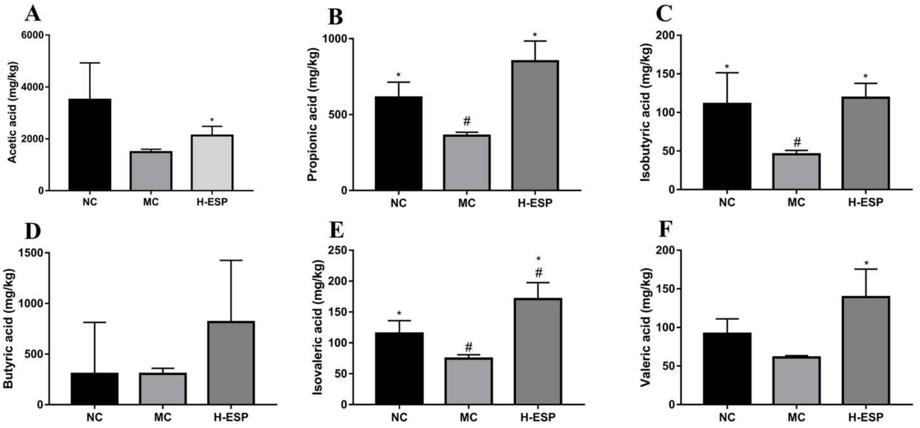

Figure S1. Effects of short-chain fatty acids of intestinal contents. A: acetic acid; B: propionic acid; C: isobutyric acid; D: butyric acid; E: isovaleric acid; F: valeric acid. (Compared with NC group, $^{\#}p$<0.05; compared with MC group, $^{*}p$<0.05).
